# Core–Shell Structured Fluorescent Protein Nanoparticles: New Paradigm Toward Zero‐Thermal‐Quenching in High‐Power Biohybrid Light‐Emitting Diodes

**DOI:** 10.1002/advs.202300069

**Published:** 2023-04-04

**Authors:** Mattia Nieddu, Marta Patrian, Sara Ferrara, Juan Pablo Fuenzalida Werner, Fabian Kohler, Eduardo Anaya‐Plaza, Mauri A. Kostiainen, Hendrik Dietz, Jesús Rubén Berenguer, Rubén D. Costa

**Affiliations:** ^1^ Chair of Biogenic Functional Materials Technical University of Munich Schulgasse, 22 94315 Straubing Germany; ^2^ Laboratory for Biomolecular Nanotechnology Department of Physics Technical University of Munich Am Coulombwall 4a 85748 Garching Germany; ^3^ Munich Institute of Biomedical Engineering Technical University of Munich Boltzmannstraße 11 5748 Garching Germany; ^4^ Department of Bioproducts and Biosystems School of Chemical Engineering Aalto University Kemistintie 1 Espoo 02150 Finland; ^5^ Departamento de Química‐Centro de Investigación en Síntesis Química (CISQ) Universidad de La Rioja Madre de Dios 53 Logroño E‐26006 Spain

**Keywords:** biohybrid light‐emitting diodes, fluorescent protein, hybrid protein‐metal oxide nanoparticles, photon downconverting filters, protein‐based lighting

## Abstract

Stable and efficient high‐power biohybrid light‐emitting diodes (Bio‐HLEDs) using fluorescent proteins (FPs) in photon downconverting filters have not been achieved yet, reaching best efficiencies of 130 lm W^−1^ stable for >5 h. This is related to the rise of the device temperature (70–80 °C) caused by FP‐motion and quick heat‐transmission in water‐based filters, they lead to a strong thermal emission quenching followed by the quick chromophore deactivation via photoinduced H‐transfer. To tackle both issues at once, this work shows an elegant concept of a new FP‐based nanoparticle, in which the FP core is shielded by a SiO_2_‐shell (FP@SiO_2_) with no loss of the photoluminescence figures‐of‐merit over years in foreign environments: dry powder at 25 °C (ambient) or constant 50 °C, as well as suspensions in organic solvents. This enables the preparation of water‐free photon downconverting coatings with FP@SiO_2_, realizing on‐chip high‐power Bio‐HLEDs with 100 lm W^−1^ stable for >120 h. Both thermal emission quenching and H‐transfer deactivation are suppressed, since the device temperature holds <40 °C and remote high‐power Bio‐HLEDs exhibit final stabilities of 130 days compared to reference devices with water‐based FP@SiO_2_ (83 days) and FP‐polymer coatings (>100 h). Hence, FP@SiO_2_ is a new paradigm toward water‐free zero‐thermal‐quenching biophosphors for first‐class high‐power Bio‐HLEDs.

## Graphical abstract

The authors prepare the first FP@SiO_2_ nanoparticles for biohybrid light‐emitting diode (Bio‐HLED) application, realizing a strong improvement of the stability in on‐chip high‐power Bio‐HLEDs thanks to the suppression of thermal emission quenching and H‐transfer deactivation. Hence, FP@SiO_2_ is a new paradigm toward water‐free zero‐thermal‐quenching biophosphors for first‐class high‐power Bio‐HLEDs.

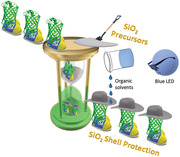

## 1 Introduction

Biohybrid light‐emitting diodes (Bio‐HLEDs) are a new class of lighting devices that combine highly efficient UV‐/blue‐emitting inorganic LED chips, as a pumping source, with sustainability and biocompatibility of photon downconverting filters, or biophosphors, based on biogenic emitters and/or packaging matrices.^[^ ^1^, ^2^, ^3^, ^4^, ^5^, ^6^ ^]^ The aim of this emerging concept is to replace the current inorganic phosphors that are based on rare earth and/or toxic emitters that put into a burn the long‐term sustainability and viability of the current LED technology as recycling protocol are inefficient and natural resources are limited.^[^ ^1^, ^2^, ^3^, ^4^, ^5^, ^6^ ^]^ It is here where biophosphor relevance sits in.

To date, biophosphors can be divided into three families: *i)* organic dyes encapsulated in biogenic matrices (e.g., deoxyribonucleic acid,^[^ ^7^ ^]^ proteins,^[^ ^8^ ^]^ and cellulose,^[^ ^9^ ^]^ among others), which achieve luminous efficiencies <50 lm W^−1^ and lifetimes (i.e., 50% loss of the initial intensity) of a few days by using on‐chip architectures (i.e., zero distance between the biophosphor and LED emitting chip) and low‐power LED chips (i.e.,<50 mW cm^−2^ excitation), *ii)* naturally evolved^[^ ^1^, ^10^, ^11^, ^12^, ^13^, ^14^ ^]^ and artificially engineered fluorescent proteins (FPs) ^[^ ^15^, ^16^ ^]^ stabilized in water‐based polymer coatings^[^ ^1^, ^10^, ^11^, ^12^, ^13^, ^14^, ^15^, ^16^ ^]^ and aqueous solutions,^[^ ^14^ ^]^ leading to best on‐chip devices with luminous efficiencies of 130 lm W^−1^ and stabilities of >5 h or 3600 h with high‐ or low‐power LED chips, respectively,^[^ ^13^ ^]^ and *iii)* fully biogenic phosphors combining FPs as emitters and silk fibroin (SF) protein as matrix,^[^ ^17^ ^]^ realizing efficiencies of 40 lm W^−1^ and stabilities of 1.2 h or 450 h with high‐ or low‐power LED chips, respectively.

Compared to the best on‐chip low‐power HLEDs with organic phosphors based on small molecules (Table S1, Supporting Information, perylene diimide‐polymer; >700 h@130 lm W^−1^),^[^ ^18^ ^]^ conjugated polymer (BODIPYs‐polymer; >10 h@13 lm W^−1^),^[^ ^19^ ^]^ and transition metal complexes (Iridium(III) complex‐polymer with >1000 h@100 lm W^−1^;^[^ ^20^ ^]^ Iridium(III) complexes@SiO_2_‐polymer with >2000 h@1.5 lm W^−1^),^[^ ^21^ ^]^ FP‐based biophosphors are becoming a frontrunner photon downconverting filter due to i) their high photoluminescence quantum yields (PLQYs) associated to narrow (20–50 nm) rainbow emission spectra,^[^ ^2^, ^6^ ^]^ ii) the easy and low‐cost bacteria production of FPs,^[^ ^4^, ^6^ ^]^ iii) their excellent stabilities over years in polymer composites under ambient storage conditions,^[^ ^1^, ^2^, ^3^, ^4^, ^5^ ^]^ and iv) the recent advances in on‐chip low‐power device performance (>3600 h@130 lm W^−1^).^[^ ^13^ ^]^

In this context, the current challenge is how to improve the on‐chip high‐power Bio‐HLED's stability that is at best of >5 h@130 lm W^−1^ (Table S1, Supporting Information).^[^ ^13^ ^]^ Common to all the reports,^[^ ^13^, ^17^, ^18^, ^19^, ^21^ ^]^ the low stability in high‐power HLEDs is attributed to the raise of the working device temperature above 70 °C. In Bio‐HLEDs,^[^ ^12^, ^13^ ^]^ this is caused by efficient heat generation and transfer processes, related to FP‐motion (vibrational/rotational) upon continuous excitation and the excess of water present in the FP‐polymer composites, respectively. This leads to a strong temperature dependent emission quenching (≈80% of the initial emission intensity in the first seconds) that is followed by an irreversible deactivation of the chromophore toward its nonemissive neutral form via protonation process (>3 h to reach 50% of the emission intensity after the first thermal quenching).^[^ ^12^, ^13^ ^]^ Recently, we have reduced the working temperature down to 40 °C (i.e., a mild thermal quenching process of ≈20%) by increasing the stiffness of the FP‐polymer composites.^[^ ^13^ ^]^ Unfortunately, this only resulted in a slightly enhanced device stability (≈5 h) as the photoinduced H‐deactivation mechanism of the protein chromophore was not affected due to excess of water surrounding the FP surface.^[^ ^13^ ^]^

Herein, we hypothesized that the encapsulation of a FP core by growing a SiO_2_ shell around (FP@SiO_2_) could simultaneously restrict protein motion and the access to the excess of water molecules surrounding the protein surface, reducing both, heat generation/transfer and photoinduced H‐transfer deactivation processes. On this basis, this work describes at first a novel synthetic method (i.e., supramolecular protein modification of carboxylic acid by 3‐aminopropyltriethoxysilane (APTES), followed by one‐pot sol‐gel chemistry) to shield superfolder green fluorescent proteins (sfGFP) core by a spherical SiO_2_ shell (i.e., sfGFP@SiO_2_ nanoparticle). This material retains the excellent emissive properties of the FPs in dry storage at 25 °C (ambient) and constant 50 °C in air over a year. Much more relevant, the photoluminescent features surprisingly hold in toluene and dichloromethane suspensions over a year. Compared to the prior‐art, these findings nicely highlight the relevance of the sfGFP@SiO_2_ design, since the biofunctionality of FPs in foreign solvent environments has only been preserved for a few days using random heteropolymers.^[^ ^22^ ^]^ What is more, the excellent stability in organic solvents allowed us for a direct comparison of the thermal and degradation behaviors of on‐chip high‐power Bio‐HLEDs with water‐based and water‐free sfGFP@SiO_2_‐polymer, as well as reference water‐based sfGFP‐polymer photon downconverting filters. While all of them exhibited excellent luminous efficiencies of ≈100 lm W^−1^ as their photoluminescence features are similar, the headlines for the device stability are: i) heat generation/transfer processes are strongly reduced (<40 °C) using water‐free biophosphors, leading to a reduced initial thermal emission quenching, and ii) photoinduced H‐deactivation is significantly slowed down by the synergistic effect of the SiO_2_ shielding (≈2‐fold enhanced device stability: water‐based sfGFP‐polymer (3 h) versus water‐based sfGFP@SiO_2_‐polymer (7 h) filters) and the water‐free environment (i.e., dichlorometane‐based sfGFP@SiO_2_‐polymer filters), achieving a device stability regime of >120 h compared to the best performing on‐chip high‐power Bio‐HLEDs operating at the same conditions (>5 h).^[^ ^13^ ^]^ This synergistic effect toward enhanced device stability is further confirmed in high‐power Bio‐HLEDs with remote architectures (i.e., the biophosphor is placed 2 cm above the emitting LED chip) that featured stabilities over 40/130 days at <10%/50% loss of emission intensity that are almost two orders of magnitude higher than the reference remote high‐power devices with water‐based sfGFP‐polymer filters (>10 h/>100 h to reach 10%/50% loss, respectively).

In light of these findings and capitalizing on the well‐known sol‐gel chemistry to fine‐tune the design of core–shell structured hybrid silica nanomaterials,^[^ ^23^, ^24^, ^25^ ^]^ this work sets in a solid stepping‐stone approach in water‐free zero‐thermal quenching phosphors to meet high efficiencies and stabilities in on‐chip high‐power Bio‐HLEDs in the mid‐term.

## 2 Results and Discussion

### 2.1 Synthesis and Spectroscopical Characterization of sfGFP@SiO_2_

**Figure 1:**
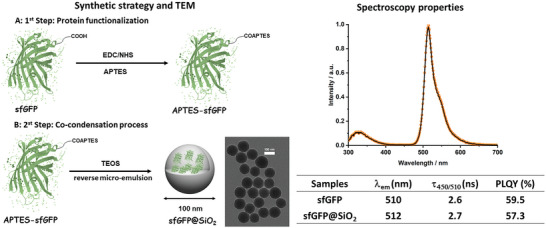
Left: Synthetic strategy and transmission electron microscopy (TEM) pictures of sfGFP@SiO_2_. Right: Emission spectra at 280 nm excitation (top) of buffer superfolder green fluorescent proteins (sfGFP) solution (solid line) and sfGFP@SiO_2_ suspension (symbol), as well as a table (below) gathering the most important figures‐of‐merit: maximum emission wavelength (*λ*_em_), excited state lifetime at selected excitation/emission (*τ*_450/510_), and photoluminescence quantum yields (PLQYs).

The sfGFP was produced following standard bacteria expression and purification procedures (Supporting Information). The sfGFP bears 35 peripheral carboxylic groups accessible for the chemical functionalization with terminal alkoxysilane groups through amidation reaction of (3‐aminopropyl)triethoxysilane (APTES) in phosphate buffer (PBS) neutral conditions upon gentle stirring overnight, leading to the functionalized APTES‐sfGFP (**Figure** **1**). These alkoxysilane groups can perform a simple cocondensation reaction with tetraethoxysilane (TEOS) by the common reversible (water‐in‐oil) emulsion method at ambient conditions (Figure 1, left). The reaction was performed by slow addition of TEOS to a mixture of the PBS solution of APTES‐sfGFP with cyclohexane and *n*‐hexanol using Triton X‐100 as surfactant. Finally, 25% ammonia was added dropwise to promote the hydrolysis and condensation of the alkoxysilane groups (supporting information). In this way, every functionalized APTES‐sfGFP is covalently embedded and tightly wrapped by the silica matrix, forming emissive sfGFP@SiO_2_.

Transmission electron microscopy (TEM) images confirm the formation of discrete nanoparticles with an average diameter of 100 nm (Figure 1, left) while elemental analysis shows 1.78% nitrogen in sfGFP@SiO_2_, corresponding to 10.2% mass ratio of sfGFP in the nanoparticle. The integration of sfGFP into the SiO_2_ network was further confirmed by the green emission of the nanoparticles that did not change to the naked eye upon extensive washing cycles using acetone, water, and ethanol (Figure S1, Supporting Information). Finally, they were characterized by means of small angle X‐ray scattering (SAXS) (Figure S2, Supporting Information). First, in absence of sfGFP, the Triton X‐100 surfactant micelles within the silica matrix shows the typical features of well‐defined spherical particles of 30.5 nm radii. In contrast, sfGFP@SiO_2_ exhibits no distinctive form‐factor features due to loss of sphericity of the micelles upon interaction with the sfGFP.

As far as the photoluminescence features are concerned, the figures‐of‐merit of the sfGFP@SiO_2_ powder and sfGFP buffer solution are the same (Figure 1, right) corroborating that neither the protein structure nor the chromophore is altered during the synthesis of the hybrid silica nanoparticles. In detail, their emission spectra at an excitation wavelength of 280 nm consist of two emission bands centered at ≈325 and 510 nm, which correspond to the fluorescence of the amino acid tryptophan (Trp 57) and the ionic form of the chromophore of the sfGFP, respectively (Figure 1, right). The peak intensity ratio (I_510_/_325_ = 5) is similar for both sfGFP and sfGFP@SiO_2_, again indicating that the protein structure is not distorted upon integration into the SiO_2_ network.^[^ ^12^, ^13^, ^26^, ^27^, ^28^, ^29^ ^]^ Likewise, the excited state lifetimes (*τ*) at each emission peak (i.e., Trp57 and sfGFP‐chromophore) and the photoluminescence quantum yield (PLQY) associated to the chromophore did not change (Figure 1, right). Finally, the lack of emission features at the 400–470 nm region attests that the chromophore did not change into its neutral form during the synthesis procedure.^[^ ^26^, ^27^, ^28^, ^29^ ^]^

**Figure 2:**
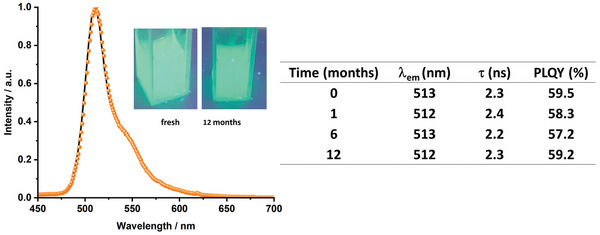
Left: Emission spectra of sfGFP@SiO_2_ at 450 nm excitation in freshly prepared (solid line) and 12‐month aged dichloromethane suspensions (symbol) along with pictures of the spectrum suspensions. Right: Table gathering the changes of *λ*_em_, *τ*_450/510_, and PLQY of sfGFP@SiO_2_ dichrolomethane suspensions over time.

While these findings clearly support the successful integration of the sfGFP into the SiO_2_ network, the beneficial effect of the SiO_2_ shielding was demonstrated upon monitoring these figures over time at room temperature and constant 50 °C storage under ambient. As shown in Figure S3 (Supporting Information), the emission profile, PLQY, and *τ* values did not change for months. In addition, the photoluminescence of the sfGFP@SiO_2_ in suspensions with organic solvents, such as toluene, and dichloromethane, surprisingly holds over a year (**Figure** **2**, and Figure S4, Supporting Information), suggesting that the interaction of solvent molecules with the protein surface through the porous structure of the SiO_2_ nanoparticle is minimal and, in turn, the environment of the chromophore cavity remains unaltered by the environmental surroundings/conditions. To date, the best strategy to solubilize and stabilize native FP derivatives and other enzymes in nonnative environments (i.e., toluene) is the design of random heteropolymers to mimic intrinsically disordered proteins that strongly interact with the protein surface forming small micelles, in which the biofunctionality of, for example, GFP derivatives is preserved for at least 24 h.^[^ ^22^ ^]^ Thus, our findings highlight that the design of core–shell structured FP@SiO_2_ is another promising route to stabilize complex biocompounds in foreign surroundings for very long periods.

### 2.2 Preparation and Characterization of High‐Power BioHLEDs

The biophosphors were prepared with an optimized polymer composition to stabilize FPs (supporting information).^[^ ^1^, ^10^, ^11^, ^12^ ^]^ In short, an aqueous solution of sfGFP (1 mg) was mixed with a low‐weight branched polyethylene oxide under stirring conditions overnight followed by a final addition of a large‐weight linear polyethylene oxide polymer in a 4:1 mass ratio, respectively. After a gentle vacuum, the reference self‐standing water‐based sfGFP‐polymer coating with a thickness of 1 mm was obtained. Likewise, biophosphors with water or dichrolomethane sfGFP@SiO_2_ suspensions were prepared adjusting for the same amount of sfGFP in both coatings of similar thickness (Supporting Information).

**Figure 3:**
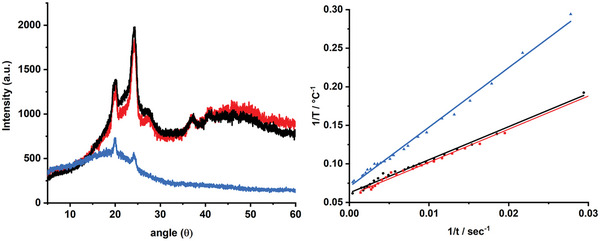
Left: X‐ray spectra of water‐based sfGFP‐polymer (black) and sfGFP@SiO_2_‐polymer (red) as well as water‐free sfGFP@SiO_2_‐polymer (blue) coatings. Right: Measurement of heat transmission speed of water‐based sfGFP‐polymer (black) and sfGFP@SiO_2_‐polymer (red) coatings (both ≈0.24 °C s^−1^) as well as water‐free sfGFP@SiO_2_‐polymer (blue) coatings (≈0.13 °C s^−1^).

As far as the characterization of the coatings is concerned, differential scanning calorimetry (DSC) data indicates that the integration of either sfGFP or sfGFP@SiO_2_ in water‐based filters does not strongly affect the thermal features (i.e., melting temperature of 50 °C with an enthalpy of 30 J g^−1^; Figure S5, Supporting Information), while those with dichloromethane showed the same melting temperature, but a reduced enthalpy (i.e., melting temperature of 50 °C with an enthalpy of 17 J g^−1^; Figure S5, Supporting Information), since the crystallinity related to the linear polyethylene oxide polymer is reduced (**Figure** **3**, left). This also results in a lower heat transmission in water‐free polymer coatings compared to those prepared from aqueous solutions (Figure 3, right). In detail, heat transfer measurements (Supporting Information) showed that water‐based filters featured a twofold higher heat transfer speed, which is well justified by the lower specific heat capacity of water (75 J mol^−1^K^−1^) compared to dichloromethane (102 J mol^−1^K^−1^) and the higher polymer crystallinity that enhances heat transfer between solvent molecules.^[^ ^30^ ^]^ Thus, an enhanced thermal behavior is expected in the devices —vide infra. Finally, by excitation at 280 nm, both sfGFP@SiO_2_ based biophosphors showed similar emission spectra (i.e., emission peaks at 510 nm and *I*_510/325_ = 5), along with the same *τ*_450/510_ and PLQY figures as those observed for sfGFP‐polymer coatings (Figure S6, Supporting Information), suggesting that the luminous efficiency should be similar for the devices—vide infra.

**Figure 4:**
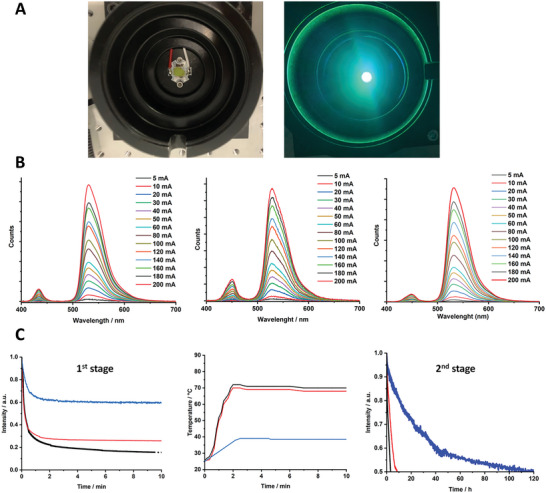
A) Pictures of water‐free sfGFP@SiO_2_ biohybrid light‐emitting diodes (Bio‐HLEDs) at work. B) Emission spectra of reference water‐based sfGFP‐polymer devices (left), as well as water‐based (middle) and water‐free (right) sfGFP@SiO_2_‐polymer devices at different applied currents. C) Device stability (left, and right) and thermal behavior (middle) of water‐based sfGFP‐polymer (black), water‐based sfGFP@SiO_2_‐polymer (red), and water‐free sfGFP@SiO_2_‐polymer (blue) on‐chip high power Bio‐HLEDs driven at constant 200 mA or 200 mW cm^−2^. The device stability is highlighted by two regimes, that is, a first exponential decay (left) over first minutes and a second linear decay over hours (right).

Next, we turned to validate our initial hypothesis on simultaneously limiting heat generation/transfer and photoinduced H‐deactivation by the silica shell. To this end, Bio‐HLEDs were fabricated using blue‐emitting LED chips (450 nm; 1 W), in which the above biophosphors were placed in on‐chip configuration (Supporting Information). The device characterization first consists in monitoring the biophosphor conversion efficiency changes of the photon downconverting emission band and luminous efficiency upon increasing the applied currents from 5 to 200 mA (**Figure** **4**B and Figure S7, Supporting Information).^[^ ^1^, ^2^, ^3^, ^4^, ^5^, ^6^ ^]^ Regardless of the type of coating, the blue‐to‐green conversion is almost quantitative for the applied current range (>90%; Figure 4), corresponding to a green‐emitting device with dominant wavelength emission at 510 nm associated to *x*/*y* CIE color coordinates of 0.30/0.65 and color purity of ≈85 (Figure 4B). In addition, the response of the emission intensity upon increasing the applied current for both LED emitting chip and downconverting emission band is linear, indicating the lack of nonlinear effects at this photon flux excitation regime – Figure S8 (Supporting Information).^[^ ^1^, ^2^, ^3^, ^4^, ^5^, ^6^ ^]^ Finally, maximum luminous efficiency values of ≈100 lm W^−1^ at 10–120 mA that slightly reduces at high applied currents due to a reduction of the internal quantum efficiency of the LED emitting chip were noted (Figure S7, Supporting Information). Thus, all the devices exhibit the same performance allowing a fair comparison between water‐based sfGFP‐polymer and sfGFP@SiO_2_‐polymer versus water‐free sfGFP@SiO_2_‐polymer filters.

Both, the thermal and stability behaviors were studied using a high applied current of 200 mA (200 mW cm^−2^ photon flux excitation), monitoring the changes in the downconverting emission band intensity and the temperature of the filter over time. Right after switching on the devices, those prepared with water‐based sfGFP‐polymer and sfGFP@SiO_2_‐polymer biophosphors show the same pattern with a first exponential decay of the emission intensity of ≈80% of the initial intensity as the working temperature quickly rises up to 70 °C in ≈100 s (Figure 4C). Thus, the SiO_2_ network does not efficiently restrict nonradiative vibrational relaxation of the sfGFP and, in turn, heat transfer to solvent molecules upon continuous excitation. This is expected as both compounds and their respective coatings featured similar *τ*, PLQY, and heat transfer speed values (Figures 1 and 3). In addition, sfGFP@SiO_2_ structure also allows heat exchange at both inside the porous at the SiO_2_‐solvent interface and the nanoparticle surface. A decrease of the pore size using different precursors (e.g., cationic, anionic, and neutral templates as well as different solvent ratios) could be beneficial to reduce heat transfer.^[^ ^23^, ^24^, ^25^ ^]^As expected by the thermal and morphological features (Figure 3), devices prepared with water‐free sfGFP@SiO_2_‐polymer coatings feature a significantly reduced thermal quenching of only 40% associated to a rise of the working temperature up to 38 °C after ≈140 s (Figure 4C).

**Figure 5:**
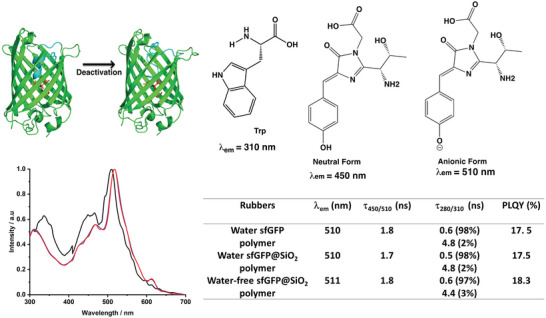
Photophysical characterization of dead biophosphors applied to on‐chip high‐power biohybrid light‐emitting diodes (Bio‐HLEDs) driven at 200 mA or 200 mW cm^−2^. Top: Schematic representation of the protein structural distortion (left) that promotes the deactivation of the emissive ionic form to the nonemissive neutral form of the chromophore (right). Bottom: Emission spectra at 280 nm excitation (left) of the water‐based sfGFP‐polymer (black) and sfGFP@SiO_2_‐polymer (red), as well as water‐free sfGFP@SiO_2_‐polymer (blue) and table gathering the most relevant figures‐of‐merit (right).

In line with the excellent thermal behavior, the device stability of the water‐free Bio‐HLEDs is also significantly enhanced after the initial thermal quenching process (Figure 4). Devices with both, water‐based sfGFP‐polymer and sfGFP@SiO_2_‐polymer coatings, exhibit a linear emission intensity decay (Figure 4C), reaching 50% of the emission intensity after thermal quenching at 3 and 7 h, respectively. The steady‐state and time‐resolved emission spectroscopy analysis show that the *τ* of the Trp57 is increased (**Figure** **5**), suggesting that the protein structure is distorted and the energy transfer from the Trp57 to the chromophore is reduced.^[^ ^26^, ^27^, ^28^, ^29^ ^]^ This typically leads to the formation of the neutral form of the chromophore as the new emission band located at 400–450 nm attested, while the *τ* and PLQY of the ionic chromophore are significantly reduced (Figure 5).^[^ ^24^, ^25^, ^26^, ^27^ ^]^ Thus, comparing both water‐based Bio‐HLEDs, the SiO_2_ shielding seems to be effective in slowing down the irreversible formation of the neutral form of the chromophore via photoinduced protonation, as the working temperature is similar in both devices. In stark contrast, devices with the water‐free sfGFP@SiO_2_ biophosphors (Figure 4C, right) show a very slow linear decay, reaching a stability regime of >120 h that are the best reported so far in on‐chip high‐power Bio‐HLEDs (>5 h at the same working conditions).^[^ ^13^ ^]^ Here, the degradation mechanism seems similar to that described in water‐based coatings (Figure 5), that is, a partial denaturation of the native structure (increase of *τ* related to Trp 57) that leads to a change in the H‐chain across the protein skeleton, promoting the formation of the neutral form (appearance of a new emission band at 450 nm in concert with decreased *τ* and PLQY values of the ionic form.) This H‐chain might include the polar amino acid groups (arginine and histidine) as well as structural water molecules that are entrapped during the formation of APTES‐sfGFP core seed (Figure 1).^[^ ^26^, ^27^, ^28^, ^29^ ^]^

**Figure 6:**
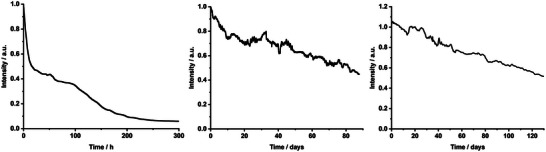
Device stability of remote high‐power biohybrid light‐emitting diodes (Bio‐HLEDs) with water‐based sfGFP‐polymer (left) and sfGFP@SiO_2_‐polymer (middle), as well as water‐free sfGFP@SiO_2_‐polymer (right) coatings at 200 mA or 200 mW cm^−2^.

To further corroborate the synergistic benefits of combining SiO_2_ shielding and water‐free environment on the stability of high‐power Bio‐HLEDs, we investigated a remote configuration, in which the working temperature holds <30 °C for all the devices (**Figure** **6**). These devices with water‐based sfGFP‐polymer coatings show a typical reduction of the luminous efficacy down to ≈10 lm W^−1^ and a step‐wise decay of the emission intensity,^[^ ^12^ ^]^ in which the first decay is associated to a partial dehydration of the protein surface, promoting protein distortion, that holds over 70 h in a plateau. This is finally followed by a second decay related to the irreversible formation of the neutral form of the chromophore.^[^ ^12^ ^]^ This accounts for stability values of >10 and *>*100 h to reach 10% and 50% loss of the initial emission intensity, respectively. Devices with water‐based sfGFP@SiO_2_‐polymer coatings feature a slow linear decay of the emission intensity (≈20% loss) over the first 300 h, holding in a plateau (<10% intensity change) for 1300 h (Figure 6). After having worked for 1600 h or 67 days, the emission intensity starts a slow linear decay, reaching a lifetime of 83 days. Though these values are notable, those with the water‐free sfGFP@SiO_2_‐polymer coating outperform them and are competitive with the best reported to date.^[^ ^13^, ^18^ ^]^ In short, the first initial decay is not present, holding in a plateau (<10% emission intensity change) for over 40 days without modifying the photoluminescence features of the coatings (Figure 6 and Figure S9, Supporting Information). This is followed by a slow linear decay, in which the chromophore evolves to its neutral form, reaching a final lifetime value of 130 days. This finding nicely pinpoints the benefits of designing FP@SiO_2_ to extraordinarily stabilize FPs in organic solvents and water‐free coatings for photonics, in general, and protein‐based lighting, in particular.

## 3 Conclusion

This work sets in a new concept toward water‐free zero‐thermal quenching biophosphors using an elegant design of a FP‐based nanomaterial, in which the sfGFP core is shieled by a SiO_2_ shell (sfGFP@SiO_2_). This protein hybrid emitter featured impressive stabilities with no loss of the photoluminescence figures‐of‐merit over years in dry powder at ambient and constant 50 °C, as well as toluene/dichoromethane suspensions. To the best of our knowledge, FP stabilities over a few days in foreign solvents have been recently realized using random heteropolymers.^[^ ^22^ ^]^ Capitalizing on the sfGFP@SiO_2_ stability, water‐based sfGFP‐polymer and sfGFP@SiO_2_‐polymer as well as water‐free sfGFP@SiO_2_‐polymer photon downconverting filters were applied to Bio‐HLEDs. While all them showed the same photoluminescence behavior, the water‐free filters featured a reduced heat transmission behavior attributed to a reduced crystallinity. This resulted in on‐chip high‐power devices operating at 38 °C with a stability regime of >120 h that represent a first‐class performance in Bio‐HLEDs (>5 h operating at the same conditions).^[^ ^13^ ^]^ This is attributed to the synergistic effect of the SiO_2_ shielding and the water‐free environment that significantly slow down the deactivation of the chromophore via H‐transfer upon continuous excitation. Indeed, remote high‐power Bio‐HLEDs featured a <10/50% loss intensity after 40/130 days that competitive with the prior‐art^[^ ^13^, ^18^ ^]^ and the reference devices with FP – i.e., 10/100 h, respectively.

In view of these findings and in concert with the myriads of chemical redesigns in hybrid silica nanomaterials,^[^ ^23^, ^24^, ^25^ ^]^ this is an important contribution toward photon downconverting filters featuring a zero‐thermal quenching and high photostability in on‐chip high‐power Bio‐HLEDs.

## Conflict of Interest

The authors declare no conflict of interest.

## Supporting information

Supporting Information

## Data Availability

The data that support the findings of this study are available from the corresponding author upon reasonable request.
