# Supplementary material for: Core–Shell Structured Fluorescent Protein Nanoparticles: New Paradigm Toward Zero‐Thermal‐Quenching in High‐Power Biohybrid Light‐Emitting Diodes
Source: Adv Sci (Weinh). 2023 Apr 4;10(16):2300069. doi: 10.1002/advs.202300069 (PMC10238177; doi:10.1002/advs.202300069)
Supplement: Supplementary file 1 — Supporting Information [file ADVS-10-2300069-s001.pdf]

ADVANCED SCIENCE Open Access

Supporting Information

for *Adv. Sci.*, DOI 10.1002/advs.202300069

Core–Shell Structured Fluorescent Protein Nanoparticles: New Paradigm Toward Zero-Thermal-Quenching in High-Power Biohybrid Light-Emitting Diodes

*Mattia Nieddu, Marta Patrian, Sara Ferrara, Juan Pablo Fuenzalida Werner, Fabian Kohler, Eduardo Anaya-Plaza, Mauri A. Kostiainen, Hendrik Dietz, Jesús Rubén Berenguer and Rubén D. Costa**

Supporting Information

Core-shell-structured Fluorescent Protein Nanoparticles: New Paradigm Towards Zero-thermal-quenching in High-power Bio-hybrid Light-emitting Diodes

*Mattia Nieddu, Marta Patrian, Sara Ferrara, Juan Pablo Fuenzalida-Werner, Fabian Kohler, Eduardo Anaya-Plaza, Mauri A. Kostiainen, Hendrik Dietz, Jesús Rubén Berenguer, and Rubén D. Costa**

Experimental Section…………………………………………………………………...…….1-6

Figures……………………………………………………………………………………7-14

Experimental Section

Protein production: The synthetic DNA construct expressing **sfGFP** was ordered through Twist Bioscience and cloned within the bacterial expression vectors pET21(+). The gene of interest was inserted under the control of T7-LacO promoter and C-terminally tagged with 6x HisTag for subsequent protein purification. The DNA sequence of the construct was verified *via* Sanger sequencing (Eurofins) and is reported here (primer T7 forward was applied to the reaction mixture):

CGGCGATATAGGCGCCAGCAACCGCACCTGTGGCGCCGGTGATGCCGGCCACGATG
CGTCCGGCGTAGAGGATCGAGATCTCGATCCCGCGAAATTAATACGACTCACTATAG
GGGAATTGTGAGCGGATAACAATTCCCCTCTAGAAATAATTTTGTTTAACTTTAAGA
AGGAGATATACATATGGTGAGCAAGGGCGAGGAGCTGTTCACCGGGGTGGTGCCCA
TCCTGGTCGAGCTGGACGGCGACGTAAACGGCCACAAGTTCAGCGTGCGCGGCGAG
GGCGAGGGCGATGCCACCAACGGCAAGCTGACCCTGAAGTTCATCTGCACCACCGG
CAAGCTGCCCGTGCCCTGGCCCACCCTCGTGACCACCCTGACCTACGGCGTGCAGTG
CTTCAGCCGCTACCCCGACCACATGAAGCGCCACGACTTCTTCAAGTCCGCCATGCC
CGAAGGCTACGTCCAGGAGCGCACCATCAGCTTCAAGGACGACGGCACCTACAAGA
CCCGCGCCGAGGTGAAGTTCGAGGGCGACACCCTGGTGAACCGCATCGAGCTGAAG

GGCATCGACTTCAAGGAGGACGGCAACATCCTGGGGCACAAGCTGGAGTACAACTT
CAACAGCCACAACGTCTATATCACCGCCGACAAGCAGAAGAACGGCATCAAGGCCA
ACTTCAAGATCCGCCACAACGTGGAGGACGGCAGCGTGCAGCTCGCCGACCACTAC
CAGCAGAACACCCCCATCGGCGACGGCCCCGTGCTGCTGCCCGACAACCACTACCT
GAGCACCCAGTCCGTGCTGAGCAAAGACCCCAACGAGAAGCGCGATCACATGGTCC
TGCTGGAGTTCGTGACCGCCGCCGGGATCACTCACGGCATGGACGAGCTGTACAAG
AAGCTTGCGGCCGCACTCGAGCACCACCACCACCACCACTGAGATCCGGCTGCTAA
CAAAGCCCGAAAGA

The DNA sequence reported here directly translates to the below amino acid sequence. A Blast protein alignment (https://blast.ncbi.nlm.nih.gov/Blast.cgi) between our amino acid sequence and the sequence reported by the creators of sfGFP[1] confirmed the identity of **sfGFP**. This protein was produced in *Escherichia coli* BL21(DE3). The cultures were induced with Isopropyl-β-D-1-thiogalactopyranoside (IPTG) at OD_{600} = 0.4. Cells were harvested, yielding a green pellet and disrupted *via* sonication (Amplitude 80, pulse on-time: 1sec, off-time 3secs). After centrifugation, the supernatant yielding the protein was purified using a HisTrap^{TM} HP 5ml column of Äkta pure cytiva, and desalted using a HiPrepTM 26/10 Desalting column (Äkta pure cytiva). After desalting, the proteins were flash-frozen in PBS buffer with a 10 mg/mL concentration and stored at -80°C. Before use, the proteins were thawed and centrifuged to remove aggregated proteins.

Sequence of ***sfGFP****:* MSKGEELFTGVVPILVELDG DVNGHKFSVR GEGEGDATNG KLTLKFICTT GKLPVPWPTL VTTLTYGVQC FSRYPDHMKR HDFFKSAMPE GYVQERTISF KDDGTYKTRA EVKFEGDTLV NRIELKGIDF KEDGNILGHK LEYNFNSHNV YITADKQKNG IKANFKIRHN VEDGSVQLAD HYQQNTPIGD GPVLLPDNHY LSTQSVLSKD PNEKRDHMVL LEFVTAAGIT HGMDELYK

Synthesis and characterization of ***sfGFP@SiO₂*** *procedures:* Chemical and materials, such as tetraethoxysilane (TEOS), (3-aminopropyl)trimethoxysilane (APTES), and Triton X-100,

were purchased from Sigma. Cyclohexane, n-hexanol, acetone and ammonia solution (GR) were obtained from Sinopharm Chemical Reagent Co., Ltd. 1-Ethyl-3-(3-dimethylaminopropyl)carbodiimide hydrochloride (EDC•HCl) and N-hydroxysuccinimide (NHS) were obtained from GL Biochem (Shanghai). The functionalization of the **sfGFP** with APTES was carried out by the conventional EDC/NHS method. Briefly, 1 mL of 10 mg/mL EGFP was mixed with 2 mL of 50 mM phosphate buffer (pH 7.5). Then 40 mg EDC•HCl and 50 mg NHS were added to the mixture and stirred for 30 min. After that, 500 μL of APTES were added, and the mixture was stirred vigorously at room temperature for 24 hours to complete the reaction. The APTES-functionalized **sfGFP** (**APTES-sfGFP**) was purified by ultra-filtration on Millipore Amicon Ultra-50 filters. The **APTES-sfGFP** was then encapsulated in silica nanoparticle (**sfGFP@SiO_2**) following the common reverse micro-emulsion method. Typically, the water-in-oil microemulsion was prepared by mixing cyclohexane (7.50 mL), Triton X-100 (1.77 mL), n-hexanol (1.80 mL), **APTES-sfGFP** solution in PBS (15 mg/mL; 300 μL), and, finally, TEOS (400 μL). The mixture was stirred at room temperature and 100 μL of a solution of 25% ammonia was added to the system to induce the condensation proccess. After stirring the mixture for 24 hours, acetone (20 mL) was added to break up the microemulsion and precipitate the **sfGFP@SiO_2** as discrete nanoparticles. They were obtained by centrifugation at 12000 rpm and washed several times with ethanol and deionized water. TEM images were obtained on a Tecnai G2 Spirit Twin (Thermo Fisher Scientific) equipped with a TemCam-F416 (TVIPS). Samples were prepared by drop-casting the particle suspension onto a carbon-coated 400 mesh copper grid and drying under room temperature. Elemental analyses were carried out in a Carlo Erba EA1110 CHNS-O microanalyzer. The samples for SAXS were prepared by confining the silica solid within a stain-steel washer between Kapton tape. The measurements were performed

using a Xenocs Xeuss 3.0 C device equipped with a GeniX 3D Cu microfocus source (wavelength λ = 1.542 Å) and EIGER2 R 1M hybrid pixel detector at a sample-detector distance of 1100 mm. Data acquisition was performed for 3× 10 min per sample. To obtain the one-dimensional SAXS data, the 2D scattering data was azimuthally averaged. The magnitude of the scattering vector q is given by q = 4πsinθ/λ with 2θ being the scattering angle. Data treatment included averaging of the triplicate 2D data of each sample, background subtraction from the Kapton tape, and Lorentz correction ($I_{Lorentz} = I \times q^2$).

Preparation and characterization of bio-phosphors: The protein-based gels are prepared as follows. As a first step, 300 µl of the respective solvent (water, acetonitrile, dichloromethane) with **sfGFP@SiO$_2$** nanoparticles (10 mg) are mixed with a branched (120 mg) and linear poly(ethylene oxide) (30 mg) compounds – *i.e.*, trimethylolpropane ethoxylate with Mn. of 450 mol. wt. and linear poly(ethylene oxide) with M_n. of 5×10^6 mol. wt. The rubber-like mixture was dried under vacuum conditions for approximately 12 h using a vacuum ramp varying from 200 mbar to 15 mbar. The photophysical studies were carried out using a FS5 Spectrofluorometer (Edinburgh Instruments) with the SC-10 module for solid samples, the SC-30 Integrating Sphere to determine ϕ, and the time-correlated single photo-counting or TCSPC (64.3 ps pulse width) module to determine τ. The data was then adjusted to a bi-exponential decay fit using Origin Softwer. To calculate the average lifetime for each FP-coating, the following equation was used $< \tau >_0 = \frac{\int_0^x t \sum a_i \, exp\left(-\frac{t}{\tau_i}\right)dt}{\int_0^x \sum a_i \, exp\left(-\frac{t}{\tau_i}\right)dt} = \frac{\sum a_i \tau_i^2}{\sum a_i \tau_i}$;[66] where a_i (λ) is the amplitude fractions and τ_i are the lifetimes. The measurements were performed at room temperature. The temperature increase was studied by placing them in a glass reservoir irradiated with a blue laser (450 nm), while recording the temperature of the gel with a thermographic camera (T430sc; FLIR). Calorimetric

essays for characterization (DSC) were carried out using a Q200 DSC from TA Instruments. Absorcion spectra were acquired with a UV-vis spectrometer UV-2600 (Shimadzu), using a wavelength range 200–800 nm, scan speed medium, threshold 0.01 and a slit width of 2.0. Stability studies in both powder and suspension were carried out under storage conditions (ambient temperature and moisture).

Preparation and characterization of devices: The bio-phosphors (coatings) were placed at zero (on top) or 2 cm from the 450 nm LED (Winger Electronics; 1W). The Bio-HLEDs were characterized using a Keithley 2400 as a current source, while the changes in the electroluminescence spectrum and lumens efficiency were monitored using an AVS-DESKTOP-USB2 (Avantes) in conjunction with a calibrated integrating sphere Avasphere 30-Irrad, while the changes in the FP-coating temperature were monitored using a thermographic camera T430sc (FLIR) coupled to the measuring system.

Figures

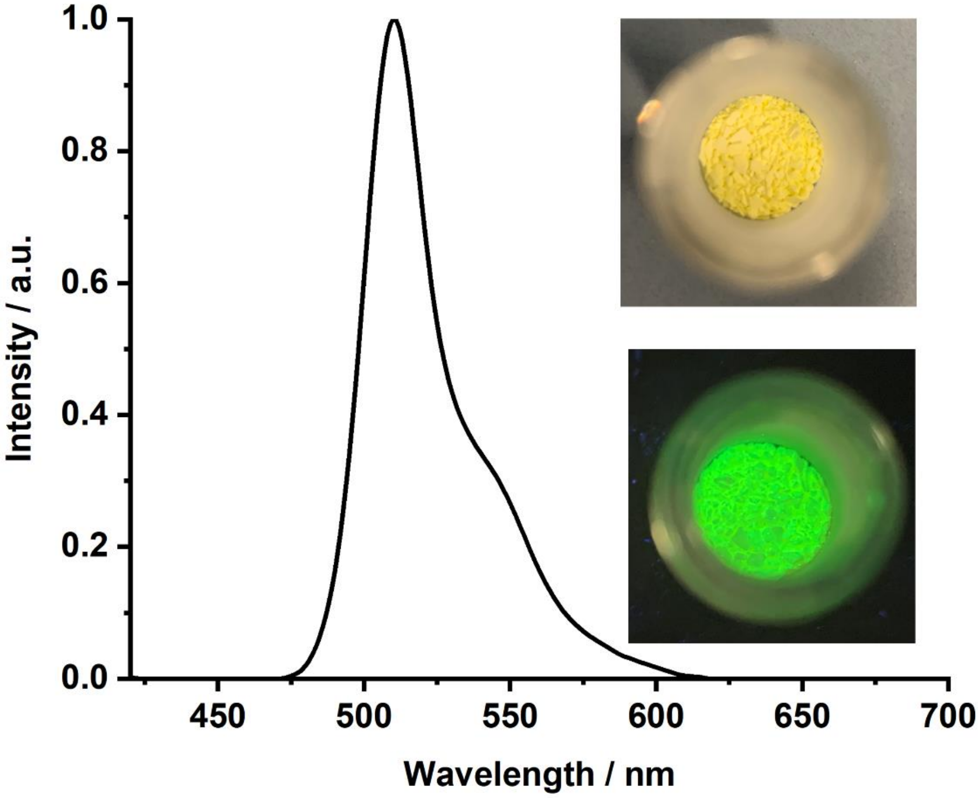

Figure S1. Emission spectra at 400 nm excitation (left) and pictures of the sfGFP@SiO_2 (right) under ambient light (top) and UV-irradiantion (bottom) after 5 washing cycles with acetone, water, and ethanol solvent.

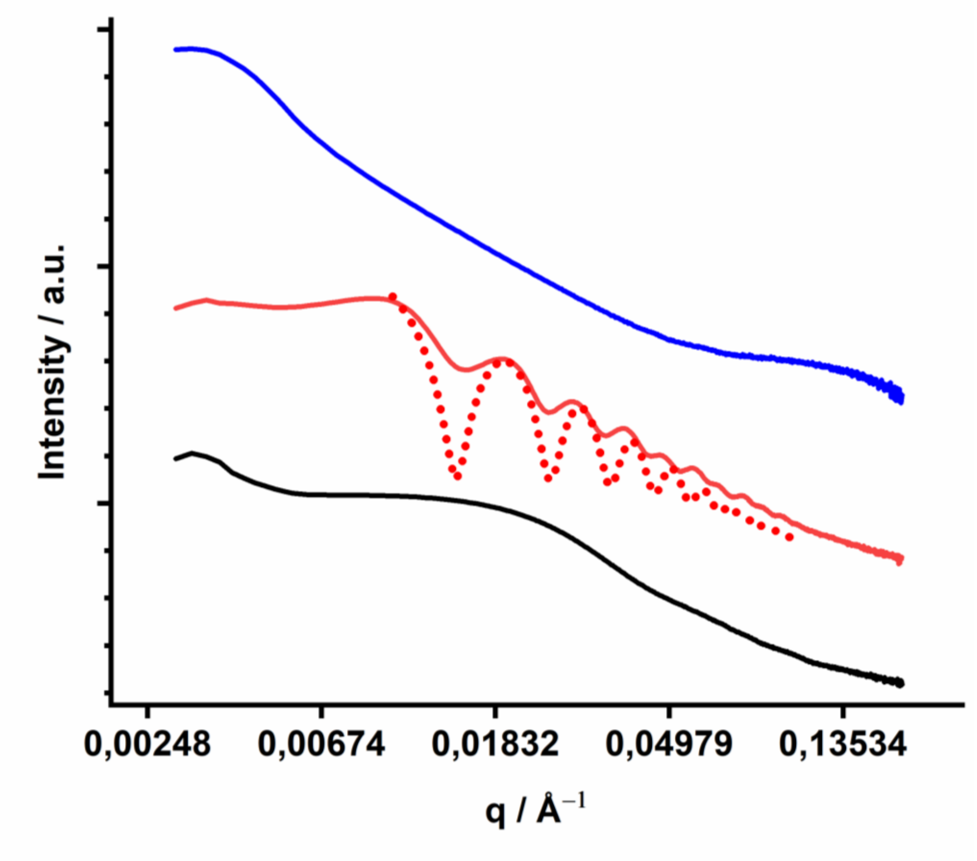

Figure S2. SAXS scattering data of amorphous SiO_2 (black), Triton X-100@SiO_2 (red), and **sfGFP@SiO_2** (blue). Scattering pattern model of solid spheres of 30.5 nm radii (red, dotted line).

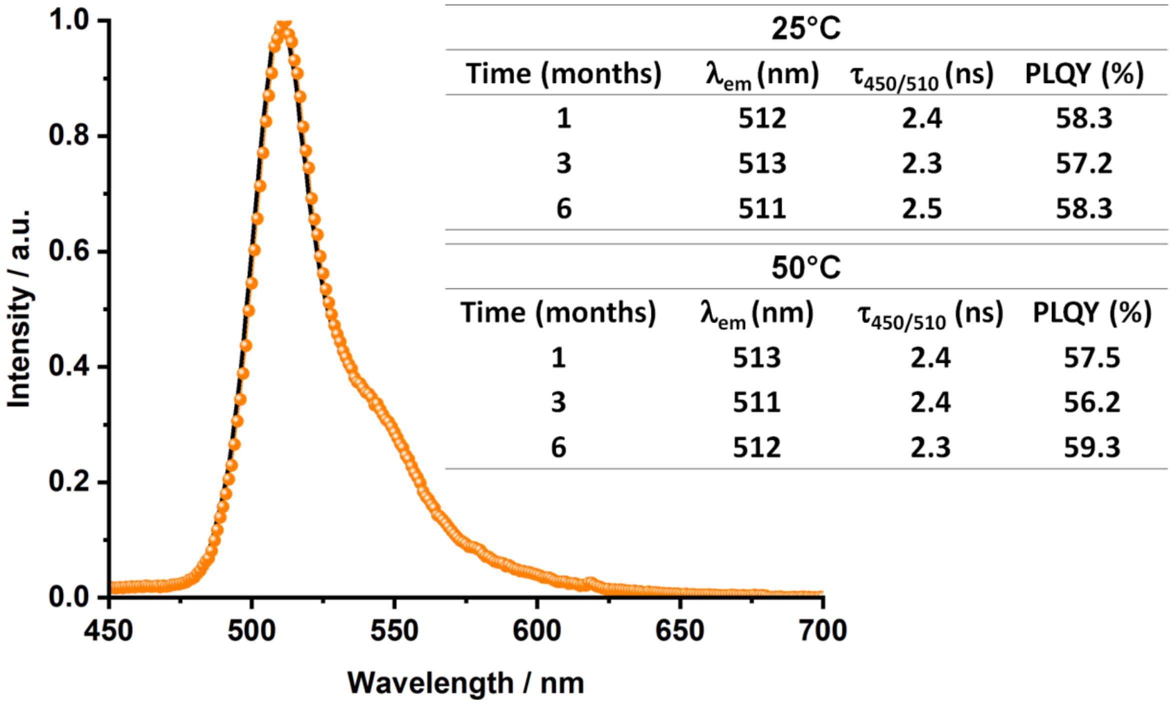

25°C			
Time (months)	λ_{em} (nm)	$\tau_{450/510}$ (ns)	PLQY (%)
1	512	2.4	58.3
3	513	2.3	57.2
6	511	2.5	58.3

50°C			
Time (months)	λ_{em} (nm)	$\tau_{450/510}$ (ns)	PLQY (%)
1	513	2.4	57.5
3	511	2.4	56.2
6	512	2.3	59.3

Figure S3. Left: Emission spectra of the freshly prepared **sfGFP@SiO_2** powder under 25°C (black), and 50°C (orange) in air. Right: Changes in λ_{em}, $\tau_{450/510}$, and PLQY of **sfGFP@SiO_2** powder over time under 25°C and 50°C in air.

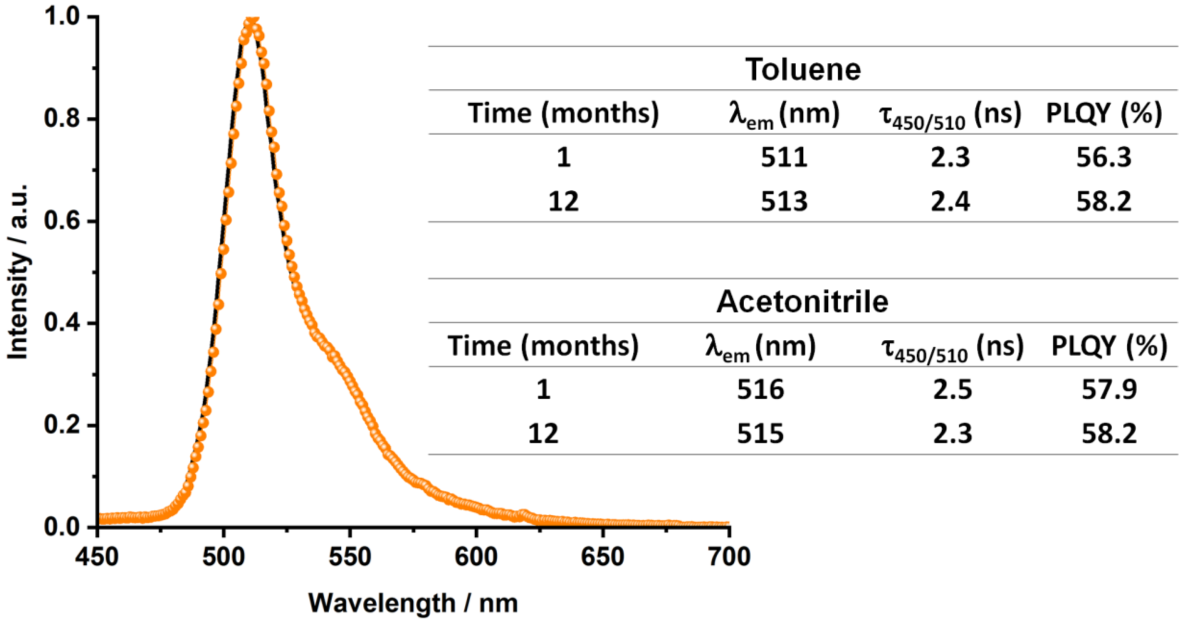

Toluene			
Time (months)	λ_{em} (nm)	$\tau_{450/510}$ (ns)	PLQY (%)
1	511	2.3	56.3
12	513	2.4	58.2

Acetonitrile			
Time (months)	λ_{em} (nm)	$\tau_{450/510}$ (ns)	PLQY (%)
1	516	2.5	57.9
12	515	2.3	58.2

Figure S4. Left: Emission spectra of **sfGFP@SiO_2** at 450 nm excitation 1 year aged suspensions prepared in toluene (orange) and acetonitrile (black). Right: Changes of λ_{em}, excited state lifetimes and PLQYs of the chromophore ($\tau_{450/510}$) of **sfGFP@SiO_2** toluene and acetonitrile suspensions over time.

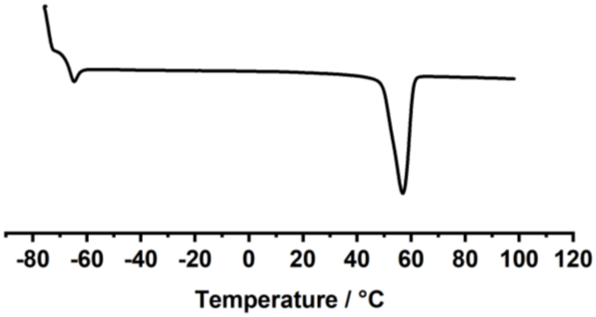

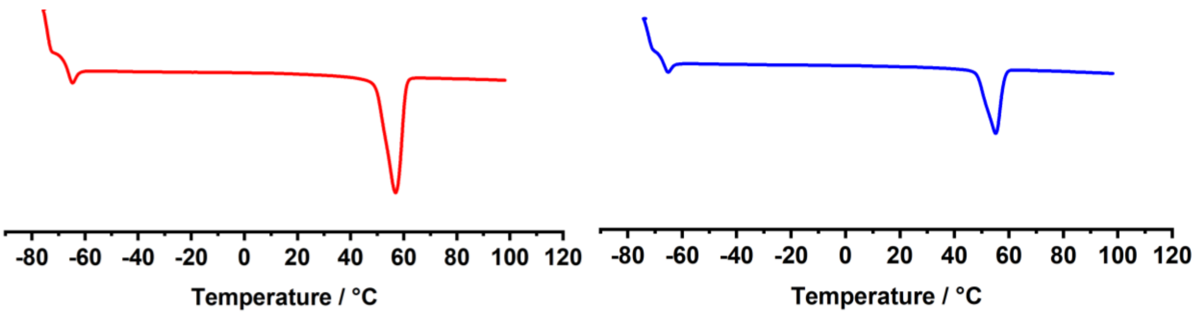

Figure S5. DSC graphs of water-based **sfGFP-polymer** (black) and **sfGFP@SiO$_2$-polymer** (red), as well as water-free **sfGFP@SiO$_2$-polymer** (blue) coatings.

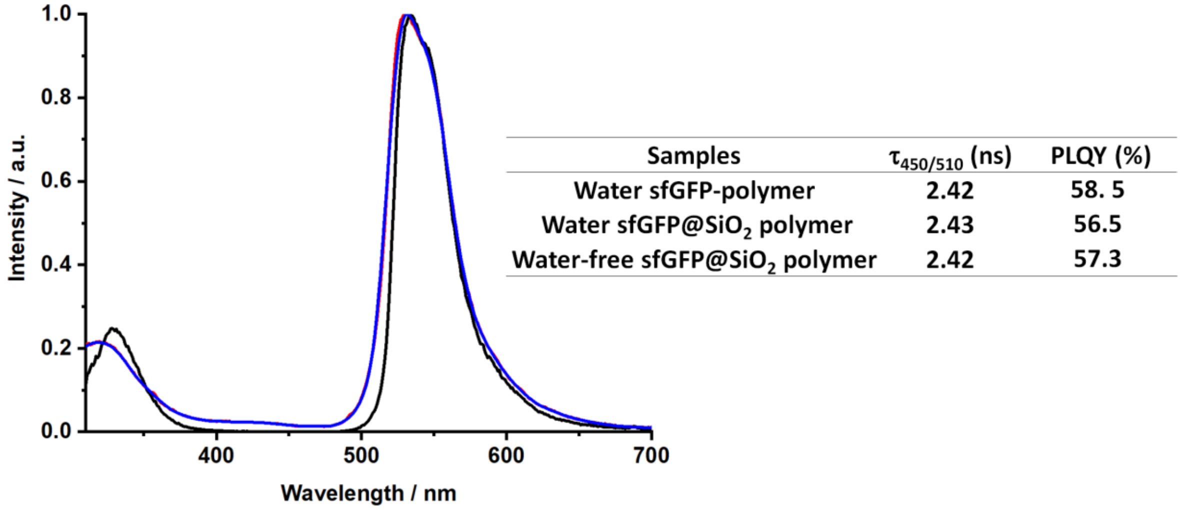

Samples	$\tau_{450/510}$ (ns)	PLQY (%)
Water sfGFP-polymer	2.42	58. 5
Water sfGFP@SiO$_2$ polymer	2.43	56.5
Water-free sfGFP@SiO$_2$ polymer	2.42	57.3

Figure S6. Emission spectra (left) and table gathering most relevant figures-of-merit (right) of water-based **sfGFP-polymer** (black) and **sfGFP@SiO$_2$-polymer** (red) coatings, as well as water-free **sfGFP@SiO$_2$-polymer** (blue) coatings.

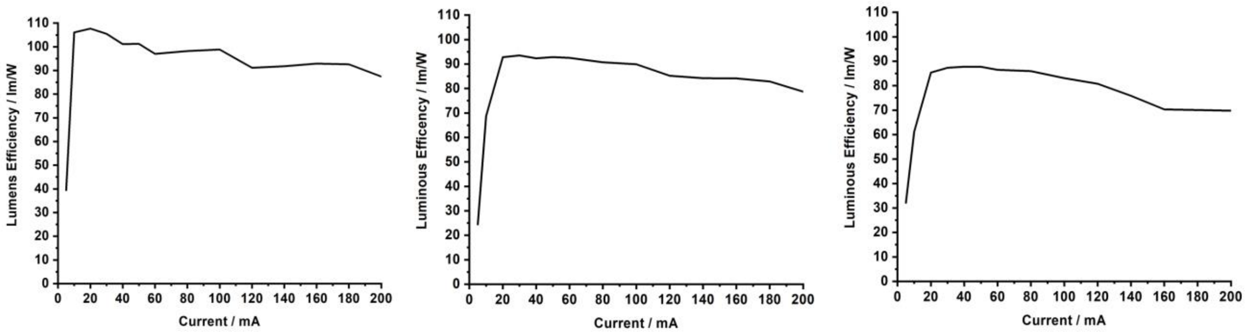

Figure S7. Changes of the luminous efficiency upon increasing the driving current from 10 to 200 mA of reference water-based **sfGFP-polymer** (left) and **sfGFP@SiO$_2$-polymer** (middle), as well as water-free **sfGFP@SiO$_2$-polymer** (right) devices.

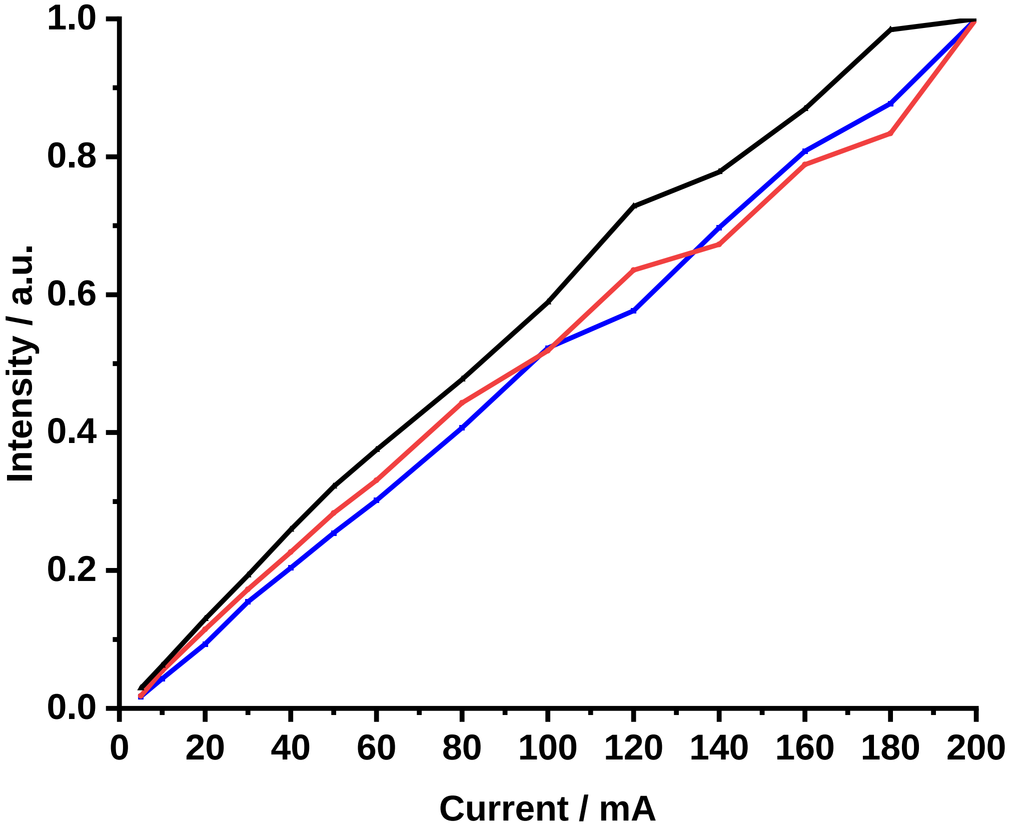

Figure S8. Change of the intensity of the down-converting emission band of Bio-HLEDs with water-based **sfGFP-polymer** (black) and **sfGFP@SiO$_2$-polymer** (red), as well as water-free **sfGFP@SiO$_2$-polymer** (blue) coatings upon increasing the applied current.

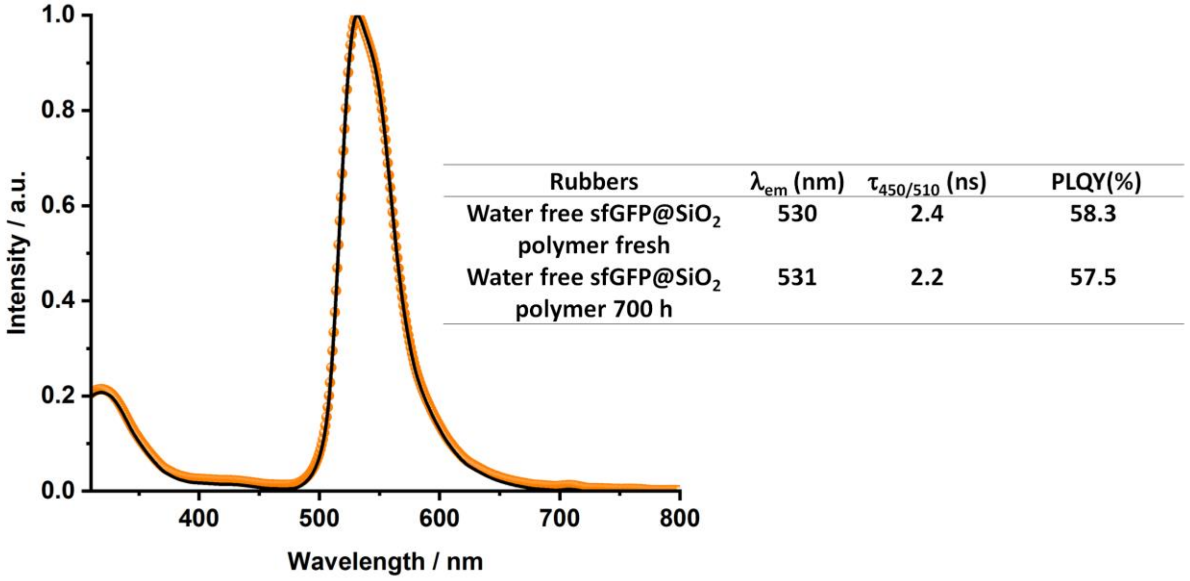

Figure S9. Emission spectra and most relevant figures-of-merit (inset table) of fresh water-free **sfGFP@SiO$_2$-polymer** coatings (black) and after 700 hours working (orange) in remote high-power Bio-HLEDs at 200 mA.

Table S1. Comparing detailed properties of on-chip HLEDs.

Pumping source	Incident Photon Flux	Luminous efficacy	Stability	Color	Reference
450 nm; - W; xx mA	-	88 lm/W	700 h	white	*Green Chem.* **2018**, *20*, 3557.
310 nm; 8W; 15 mA.	-	5 lm/W	10,000 h	white	*Mater. Horiz.,* **2019**, *6,* 13.
450 nm; - W; - mA	-	14 lm/W	28 days (discontinuous switch on for a few seconds)	white	*Adv. Mater.* **2014**, *26,* 7290.
			Bio-phosphor		
390 nm; -W; 20 mA	10 mW/cm^2	50 lm/W	50 h	white	*Adv. Mater.,* **2015**, *27,* 5493.
450 nm; - W; - mA	**-**	24 lm/W	**-**	white	*J. Appl. Nat. Sci.,* **2016**, 20.

460 nm; - W; - mA	-	81 lm/W	-	white	*Adv. Mater. Technol.* **2020**, *5*, 2000061.
450 nm; 1 W; 200 mA	200 mW/cm^2	120 lm/W	5 h	green	*Nature Commun.* **2020**, *11*, 879.
450 nm; 1 W; 200 mA	200 mW/cm^2	100 lm/W	120 h	green	Our work

Reference

1. Jean-Denis Pédelacq, Stéphanie Cabantous, Timothy Tran, Thomas C Terwilliger, and Geoffrey S Waldo *Nature Biotechnology* **2006**, *24*, 79.
